# Spatial Coherence Adaptive Clutter Filtering in Color Flow Imaging—Part II: Phantom and *In Vivo* Experiments

**DOI:** 10.1109/ojuffc.2022.3184909

**Published:** 2022-06-21

**Authors:** WILL LONG, DAVID BRADWAY, RIFAT AHMED, JAMES LONG, GREGG E. TRAHEY

**Affiliations:** 1 Philips, Cambridge, MA 02141 USA; 2 Department of Biomedical Engineering, Duke University, Durham, NC 27708 USA; 3 Department of Radiology, Duke University Medical Center, Durham, NC 27710 USA

**Keywords:** Acoustic clutter, adaptive clutter filtering, color flow imaging, image quality, spatial coherence, ultrasound

## Abstract

Conventional color flow processing is associated with a high degree of operator dependence, often requiring the careful tuning of clutter filters and priority encoding to optimize the display and accuracy of color flow images. In a companion paper, we introduced a novel framework to adapt color flow processing based on local measurements of backscatter spatial coherence. Through simulation studies, the adaptive selection of clutter filters using coherence image quality characterization was demonstrated as a means to dynamically suppress weakly-coherent clutter while preserving coherent flow signal in order to reduce velocity estimation bias. In this study, we extend previous work to evaluate the application of coherence-adaptive clutter filtering (CACF) on experimental data acquired from both phantom and *in vivo* liver and fetal vessels. In phantom experiments with clutter-generating tissue, CACF was shown to increase the dynamic range of velocity estimates and decrease bias and artifact from flash and thermal noise relative to conventional color flow processing. Under *in vivo* conditions, such properties allowed for the direct visualization of vessels that would have otherwise required fine-tuning of filter cutoff and priority thresholds with conventional processing. These advantages are presented alongside various failure modes identified in CACF as well as discussions of solutions to mitigate such limitations.

## I. INTRODUCTION

Color flow imaging has become a standard mode on virtually all clinical ultrasound scanners and is widely used for diagnosing cardiovascular abnormalities, guiding needle biopsies, and examining tumor vascularity, among many other applications [1]. In the liver, color flow images can provide valuable information unobtainable from conventional B-mode for identifying hepatocellular carcinoma and tracking the progression of liver transplantation and cirrhosis [2]–[5]. In fetal sonography, color flow imaging has become a routine component of the second trimester exam for assessing heart function and anatomy and detecting the impedance of blood flow in the umbilical cord [6], [7].

Despite its widespread use, color flow imaging remains challenged by its poor accuracy and sensitivity, particularly in the presence of tissue and transducer motion [8]–[10]. These limitations are in large part a result of sub-optimal clutter filtering – a pre-processing step applied prior to velocity estimation to remove unwanted slowly-moving, low frequency (pulse-to-pulse sampling) acoustic noise known as clutter. Clutter filters with wide passbands and low cutoff frequencies are susceptible to velocity underestimation due to poor attenuation of clutter, while more aggressive filters with higher cutoff frequencies compromise the detection of slow flow, resulting in the complete or partial removal of flow signal [11]–[13]. In one study examining color flow imaging of the fetal umbilical cord [14], clutter filter selection was noted as one of the primary sources of error in measuring volume rate in arterial and venous umbilical flow. As demonstrated by numerous studies [12], [15], such errors are clinically relevant and have been directly linked to false or missed diagnoses.

In attempt to address such issues, methods have been developed to adapt clutter filters based on measured clutter content. These techniques apply estimates of tissue motion prior to filtering to either down-mix clutter into the filter stopband [16], [17] or adjust the stopband itself to optimally attenuate clutter [18], [19]. Others apply various forms of eigen-based filtering to remove slow-time signal components based on assumed thresholds for clutter frequency and magnitude [20]–[23]. To date, virtually all existing methods derive feedback from measurements of echo magnitude and frequency or a combination of both to perform adaptive filtering. As a result, they suffer from well-known limitations when magnitude and frequency information are insufficient to separate blood and tissue backscatter from clutter [20].

In a companion paper, we introduced a novel method to adapt clutter filtering based on measurements of backscatter spatial coherence [24]. Under the assumption that clutter has lower spatial coherence than signal from on-axis tissue or blood, this method adapts clutter filtering on each frame and pixel of color flow data by applying pixel-wise measurements of spatial coherence at the output of multiple clutter filters and selecting the filter that maximizes local coherence for subsequent velocity estimation. As demonstrated by this previous study, spatial coherence is able to provide a direct measure of clutter that is inversely correlated with both velocity estimation bias from acoustic clutter and high variance jitter from thermal noise. By relying exclusively on spatial coherence feedback and without any direct assumptions related to clutter magnitude or frequency, coherence-adaptive clutter filtering or CACF was observed to provide accurate measurements of slow flow under clutter-free conditions and reduced bias under cluttered conditions in Field II simulation [25].

In the present study, we extend previous simulation results to evaluate the practical application of CACF in phantom experiments with calibrated flow rates and different clutter realizations as well as *in vivo* color flow acquisitions of liver and fetal vessels. section II provides a brief introduction to the conventional color flow processing pipeline and the modifications required to perform CACF. In section II-B, we describe the methods used to compare the performance between conventional processing and CACF. The results of these comparisons along with example matched color flow images and a discussion of the advantages and limitations of CACF compared to conventional color flow processing are presented in sections III and IV, with concluding remarks in section V.

## II. METHODS

### A. CONVENTIONAL COLOR FLOW PROCESSING

In color flow imaging, transmit-receive cycles are repeated at each imaging location to collect an ensemble of channel echo signals. Using delay-and-sum beamforming, these channel signals are time-delayed and summed to form beams that sample each imaging location in “slow-time” at intervals determined by the pulse repetition frequency (PRF).

To isolate echoes from moving blood or tissue and remove those corresponding to slow-moving or stationary clutter, each ensemble is filtered along the slow-time dimension typically using a finite or infinite impulse response (FIR or IIR) filter with a fixed frequency response. Color flow images are formed by measuring the average phase shift between clutter filtered slow-time samples. This is commonly performed using 2-D autocorrelation:

(1) $$\hat{\phi }[\vec{r}]=\tan^{-1}\left(\frac{\sum_{k=1}^{K}\sum_{r\in \Delta \vec{r}}Q[r,\;k\;+\;1]I[r,\;k]-Q[r,\;k]I[r,\;k\;+\;1]}{\sum_{k=1}^{K}\sum_{r\in \Delta \vec{r}}I[r,\;k\;+\;1]I[r,\;k]+Q[r,\;k\;+\;1]Q[r,\;k]}\right)\text{,}$$

where *I* and *Q* represent the complex in-phase and quadrature beamsum signals, respectively, acquired at slow-time samples *k* and *k* + 1 across an ensemble of *K* repeated lines for a kernel $\Delta \vec{r}$ centered about imaging location $\vec{r}$ [26]. The phase shift $\hat{\phi }$ is converted to an estimate of axial velocity, given assumptions for the sound speed *c*, center frequency *f*_0_, and PRF where:

(2) $$\hat{v}[\vec{r}]=-\frac{c\text{PRF}}{4\pi f_{0}}\hat{\phi }[\vec{r}].$$

Prior to being mapped to the output display, velocity estimates are passed through what is commonly referred to as a priority encoder, which functions to identify and display pixels containing reliable velocity information, while rejecting those corresponding to noise and clutter. Among the many methods employed in commercial systems, two common techniques for priority encoding include variance and power thresholding [27]. Variance thresholds are applied to eliminate regions of spatially and temporally random velocity estimates from thermal noise, also known as jitter, while power thresholds are applied to remove low amplitude echoes from clutter or thermal noise.

In practice, such thresholds can be implemented in the following manner where, for a given spatial kernel $\Delta \vec{r}$ centered about spatial location $\vec{r}$, the output color flow pixel at $\vec{r}$ is rejected and set to 0 cm/s if the variance inside the kernel is above some threshold *σ*^2^:

(3) $$if\;\frac{1}{\left|\Delta \vec{r}\right|}\sum_{r\in \Delta \vec{r}}{\left(\hat{v}[r]-\bar{v}\right)}^{2}>\sigma^{2}\;then\;\hat{v}[r]=0,$$

where $\bar{v}$ is the average velocity within $\Delta \vec{r}$:

(4) $$\bar{v}=\frac{1}{\left|\Delta \vec{r}\right|}\sum_{r\in \Delta \vec{r}}\hat{v}[r].$$

Additionally, the pixel is rejected if the average power of clutter filtered signals inside the kernel is below some power threshold P given in decibels:

(5) $$if\;10\;\log_{10}\left(\frac{\frac{1}{\left|\Delta \vec{r}\right|}\sum_{r\in \Delta \vec{r}}\sum_{k=1}^{K}|U[r,\;k]{|}^{2}}{\underset{\left\{r\in \text{image}\right\}}{\max}\sum_{k=1}^{K}|U[r,\;k]{|}^{2}}\right)<\text{P}\;then\;\hat{v}[r]=0\text{,}$$

where *U* are the beamsummed IQ signals at slow-time sample *k*. In the clinical workflow, these thresholds are tuned by users to balance between the appearance of artifacts and real flow.

Together, the above components make up the core processing steps used in this study to implement conventional color flow imaging:

1. Clutter filtering using standard IIR filters
2. Velocity estimation via Eqs. (1) and (2)
3. Priority encoding via Eqs. (3) to (5)

### B. COHERENCE-ADAPTIVE COLOR FLOW PROCESSING

CACF applies image quality feedback derived from backscatter spatial coherence to adaptively inform the selection of clutter filters in each frame and at each pixel in a color flow image. While CACF does not require any custom transmit sequencing, modifications in receive processing are needed to measure spatial coherence and its response to changes in clutter filtering.

**FIGURE 1.**
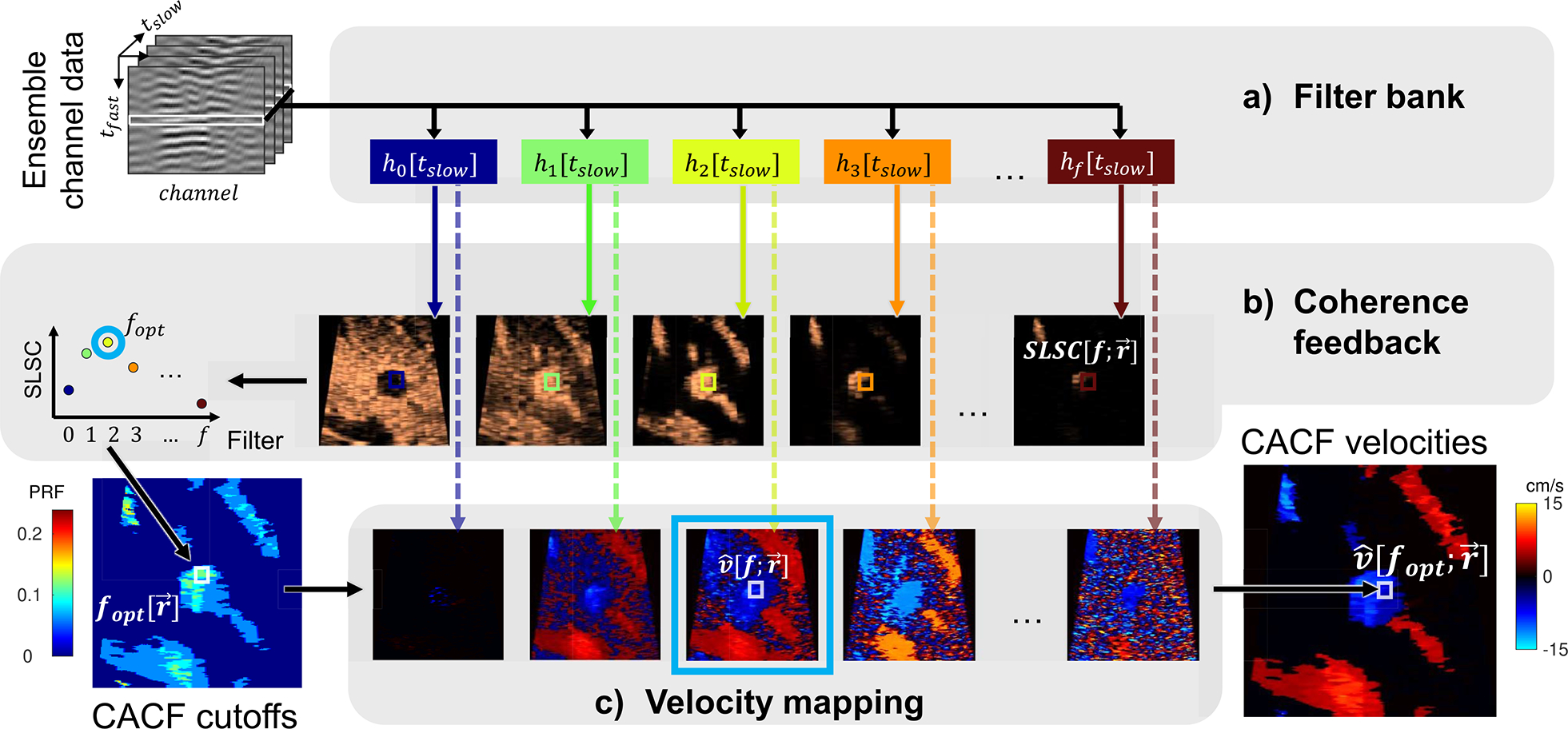
The proposed algorithm for CACF where at pixel location $\vec{r}$ (a) ensemble channel data are passed through a series of clutter filters, *h*_*f*_, (b) SLSC is measured at the output of each filter to identify the filter *f*_*opt*_ that maximizes spatial coherence, and (c) the corresponding velocity estimate, $\hat{v}\left[f_{opt};\;\vec{r}\right]$, at the output of *f*_*opt*_ is mapped to an output image. This process is repeated across all pixels to form a CACF color flow image. Note that conventional color flow imaging is roughly summarized by one vertical column of this diagram going along the dotted line from a single clutter filter to its output velocity image.

Fig. 1 describes the pipeline for color flow image formation using CACF. As in conventional color flow imaging, channel data are collected over an ensemble of slow-time samples and time-delayed to focus the received echoes. Rather than summing the channel signals prior to clutter filtering, as is done in conventional color flow imaging, clutter filtering is performed on a per-channel rather than per-beam basis to enable the measurement of spatial coherence from post-clutter filtered channel data.

In this study, the spatial coherence is calculated as the normalized correlation between channel signals averaged as a function of the spatial separation or lag *m* and across slow-time samples *k*:

(6) $$\hat{R}[m;\;\vec{r}]=\frac{1}{K(M\;-\;m)}\sum_{k=1}^{K}\sum_{i=1}^{M-m}\frac{u_{i}[\vec{r},\;k]u_{i+m}^{*}[\vec{r},\;k]}{\left|u_{i}[\vec{r},\;k]\right|\left|u_{i+m}[\vec{r},\;k]\right|}$$

where *M* is the total number of receive elements, *K* is the slow-time ensemble size, and *u*_*i*_ and *u*_*i*+*m*_ are time-delayed, clutter filtered complex IQ channel signals received at elements *i* and *i*+*m*. Note that measurements of correlation in Eq. (6) are restricted to signals obtained over the same transmit-receive cycle, and thus they have no dependence on scatterer motion.

To derive a single measurement of coherence at each imaging location $\vec{r}$, estimates from Eq. (6) are summed up to a maximum lag *Q* to compute the short-lag spatial coherence (SLSC):

(7) $$\mathrm{SLSC}[\vec{r}]=\sum_{m=1}^{Q}\hat{R}[m;\;\vec{r}].$$

To characterize color flow image quality across a bank of clutter filters, measurements of SLSC are obtained from the same channel data passed in parallel through a bank of different clutter filters. As shown in Fig. 1, adaptive filter selection is performed by comparing the spatial coherence at each imaging location $\vec{r}$ across different filters and identifying the filter *f*_*opt*_ which maximizes the spatial coherence. The velocity estimate at the output of filter *f*_*opt*_ and at $\vec{r}$ is subsequently mapped to the corresponding location in a final output image. This process is repeated across all imaging locations to form a CACF color flow image, wherein each pixel represents the output velocity of the clutter filter with the highest local spatial coherence.

### C. DATA ACQUISITION

Color flow channel data were acquired using a C5–2v curvilinear array on the Verasonics Vantage 256 system transmitting at 3.5 MHz with an 8 cm focal depth and F/2 focal geometry with rectangular apodization. Receive data were captured at a 14 MHz sampling frequency and dynamically focused with a constant F/2 rectangular apodization. At each imaging location, transmits were sampled over 14 ensemble firings at 3 kHz PRF. Each acquisition consisted of 20 full color flow imaging frames consecutively acquired at 12 Hz, giving a total acquisition time of 1.7 seconds. This acquisition sequence was used to collect all datasets in the study.

### D. PHANTOM EXPERIMENTS

To evaluate the performance of CACF under calibrated imaging conditions, a series of experiments was performed using a CIRS 069 Doppler flow phantom (Norfolk, VA). Blood was simulated using a 1% (mass/mass) starch-water mixture and circulated through a 4.8 mm diameter vessel which was oriented at a 70° Doppler angle and embedded at 8 cm depth in a tissue-mimicking material. Flow was introduced using a peristaltic pump connected to a pulse dampener to maintain continuous flow through the vessel.

Color flow data were acquired for both clutter-free and cluttered conditions by imaging through water and porcine abdominal wall, respectively. To maintain a fixed imaging window across all phantom conditions, the transducer was offset 5 cm from the top surface of the phantom using a water-filled well affixed atop the phantom. This allowed for the placement of intervening tissue while keeping the same relative positioning of the phantom and transducer. Cluttered imaging conditions were examined for two tissue samples of different thickness, consisting of intact skin, connective tissue, fat, and muscle.

To quantify the relative clutter levels generated by the two samples, the lag-one coherence (LOC) was measured from fixed 1 × 1 cm regions-of-interest (ROIs) of uniform speckle in the phantom following the methods outlined in [28]. B-mode contrast was also measured between an ROI placed in anechoic water below each abdominal wall (clutter only) and a nearby ROI inside the phantom (clutter and speckle).

**FIGURE 2.**
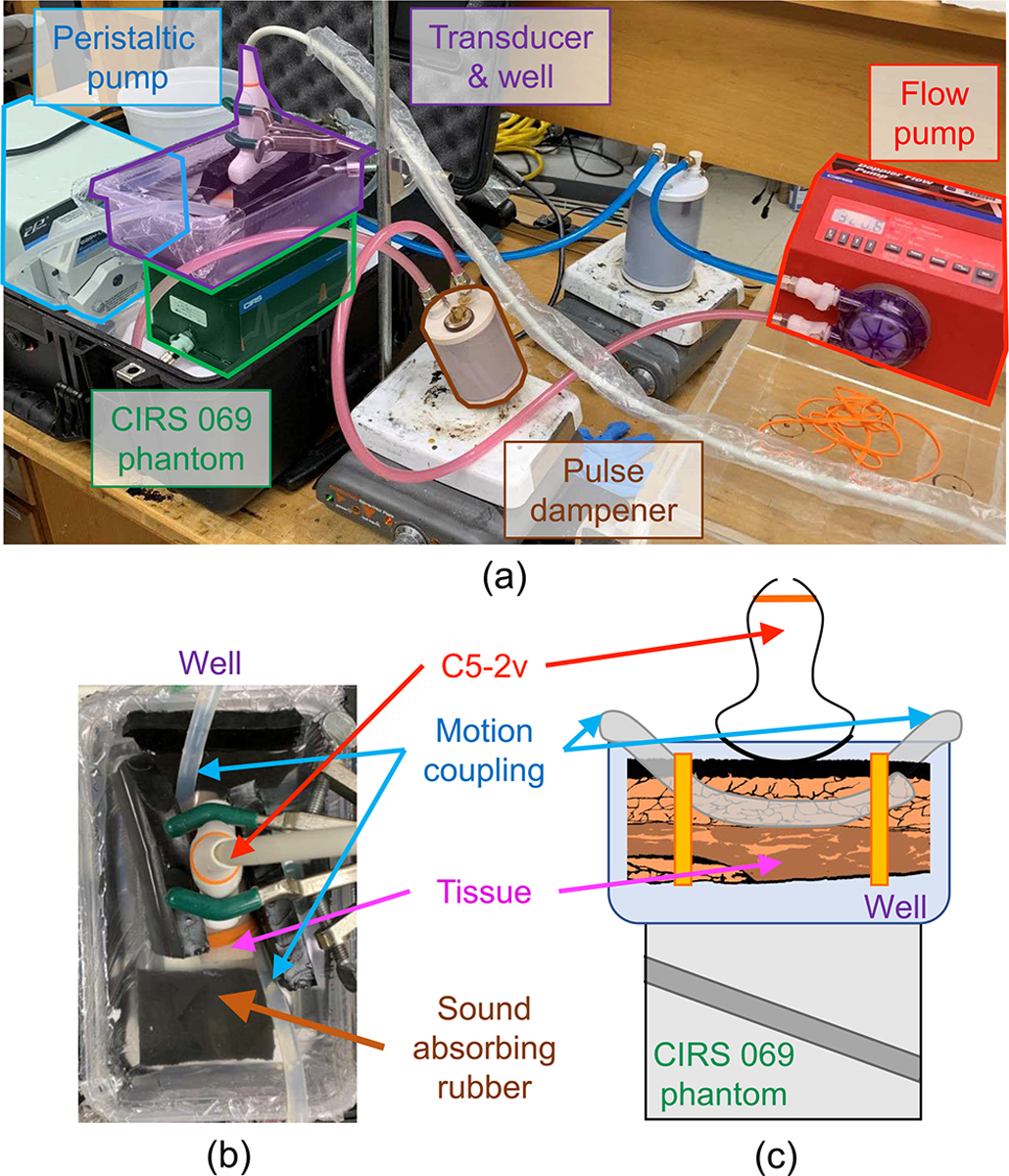
(a) Experimental setup used to image flow under conditions with moving clutter. (b) Top-down view of the well and (c) cross-sectional view of the well and phantom. Starch-water mixture is pumped through the CIRS 069 phantom and imaged using a C5–2v transducer through an intervening well containing water and a porcine abdominal layer. Using a separate peristaltic pump, motion is coupled through tubing attached to each side of the porcine abdominal layer to generate moving clutter.

Motion in the tissue layers was introduced using tubes connected to a second peristaltic pump, operated separately from the flow pump, that served to couple 2 Hz cyclic motion into each sample. Tubing was fastened to the sides of each tissue sample using elastic bands and arranged in a way that maintained a clear acoustic path between the transducer, tissue, water, and phantom. Water in the well was filled sufficiently high to submerge both the transducer face and tissue, while keeping the tissue out of direct contact with the phantom. In this way, the well served not only to maintain a fixed imaging window, but also to physically decouple tissue from the phantom, allowing the intervening tissue to move while keeping the phantom stationary. Images of the experimental setup, including top-down and cross-sectional views of the phantom and well, are shown in Fig. 2.

Color flow channel data were acquired through water and the two tissue samples for flow pump settings from 160 to 320 mL/min, corresponding to calibrated flow rates from approximately 5 to 10 cm/s after Doppler angle correction (*v* = *v*_0_ cos70°). Channel data were used to form color flow images using conventional clutter filtering with projection-initialized IIR filters with cutoff frequencies from *f*_*c*_ = 0.03 to 0.14 · PRF corresponding to velocity cutoffs *v*_*c*_ = 2.0 to 9.2 cm/s. CACF was implemented using SLSC feedback (*Q* = 10) and a filter bank consisting of 8 IIR filters with cutoff frequencies spanning from *f*_*c*_ = 0.03 to 0.24·PRF (*v*_*c*_ = 2.0 to 15.8) in addition to a “no filter” condition in which velocity and coherence estimates were measured directly from the raw channel signals without clutter filtering.

Average velocities inside the vessel were compared between conventional filtering with different cutoff frequencies and CACF. Averaging was performed over ROIs spanning the entire lateral and axial extent of the vessel and across all 20 frames to reduce the effects of residual pulsatility from the pump. Velocities obtained from the lowest cutoff filter (*f*_*c*_ = 0.03) under clutter-free imaging conditions were used as a standard of comparison between the different filter and clutter conditions. These serve to represent the “ideal” velocity estimates with minimal bias from either clutter or the attenuation of slow flow.

Average velocities in stationary regions outside the vessel were also measured to evaluate the sensitivity of conventional and coherence-adaptive filtering to flash artifacts generated by moving clutter. This analysis was repeated for conventional color flow images formed with varying levels of priority encoding with variance thresholds ranging from 0.1 to 3 mm/s and power thresholds from −35 to −10 dB, both applied over 2 × 2 mm kernels.

### E. In Vivo STUDIES

Using the same acquisition sequence as in phantom experiments, color flow channel data were acquired from *in vivo* liver and fetal vessels. Liver data were acquired from a healthy volunteer with a body mass index (BMI) of 24.4 kg/m^2^. Fetal data were acquired at the Duke Fetal Diagnostic Center from a second trimester fetus in a 21 year-old patient with a pre-pregnancy BMI of 32.1 kg/m^2^. Written consent was obtained from all study participants, and all data were collected under protocols approved by the Duke University Medical Center Institutional Review Board.

Channel data were used to form matched color flow images with conventional and coherence-adaptive filtering implemented in the same manner as in the phantom experiments. Images formed with CACF were qualitatively compared to conventional color flow images formed with different clutter filters and varying degrees of priority encoding. To evaluate the temporal and spatial stability of CACF, the average and standard deviation of velocity estimates within select vessels in the liver and fetal color flow images were measured across the 20 sequentially acquired frames in each dataset. ROIs were selected based on vessels identified from B-mode images captured prior to each color flow acquisition and used for both conventional and coherence-adaptive filtering conditions.

## III. RESULTS

### A. PHANTOM EXPERIMENTS

#### 1) CLUTTER MEASUREMENTS

Table 1 shows the measured contrast and LOC in each of the phantom imaging conditions. As expected, contrast and LOC are greatest when imaging through water and decrease with clutter from intervening tissue. Of the two samples, tissue #2 is not only thicker, but results in lower values of LOC and contrast, indicating higher levels of clutter and noise relative to tissue #1.

#### 2) COLOR FLOW ACCURACY

**FIGURE 3.**
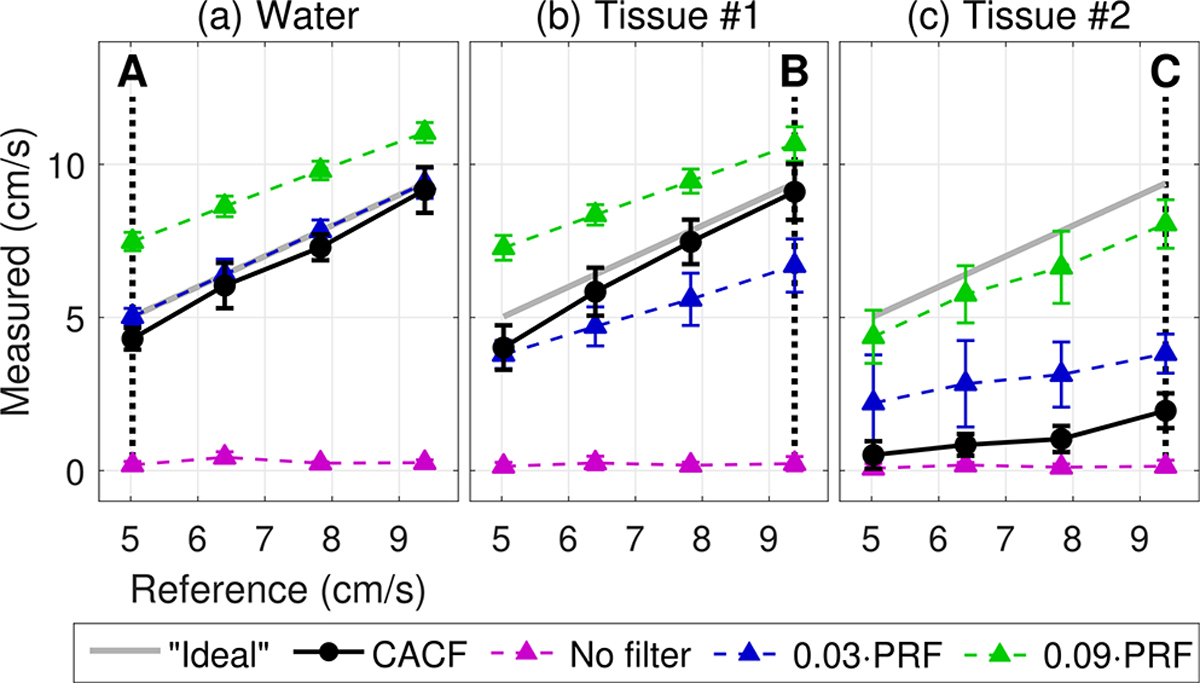
Measured average velocity inside the CIRS 069 vessel for color flow images formed with no filtering, conventional filtering with *f_c_* = 0.03 and 0.09 · PRF, and CACF plotted against the “ideal” average velocities measured using *f_c_* = 0.03 under clutter-free conditions. Data are shown for images acquired through (a) water, (b) tissue #1, and (c) tissue #2. Error bars represent the standard error across the vessel and 20 consecutively acquired frames. Points A, B, and C correspond to example images in Fig. 4a, Fig. 4b, and Fig. 4c, respectively.

Fig. 3 plots the average velocity measured inside the CIRS 069 vessel under different flow and clutter conditions for images formed with no clutter filtering, conventional filtering with low (*f*_*c*_ = 0.03 · PRF; *v*_*c*_ = 2.1 cm/s) and high (*f*_*c*_ = 0.09 · PRF; *v*_*c*_ = 5.9 cm/s) cutoff frequencies, and CACF. Average velocities are plotted as a function of “ideal” average velocities from the low cutoff filter under clutter-free conditions with the gray diagonal representing a one-to-one correspondence between the measured and ideal velocities.

Under clutter-free conditions in Fig. 3a, it follows that the low cutoff filter falls directly along the diagonal. CACF closely matches this ideal behavior, while the high cutoff filter shows an upward bias that decreases with increasing flow rate. In Fig. 3b, slow-moving clutter from tissue #1 is expected to introduce a downward bias in velocities. This is most obvious in the low cutoff filter, but less so in the high cutoff filter and CACF, which maintain roughly the same behavior as in Fig. 3a. In Fig. 3c, clutter from the thicker tissue #2 leads to severe velocity underestimation across all filters, particularly in CACF, which shows velocities well below the diagonal and on par with those measured using no filter.

#### 3) COLOR FLOW IMAGES

**FIGURE 4.**
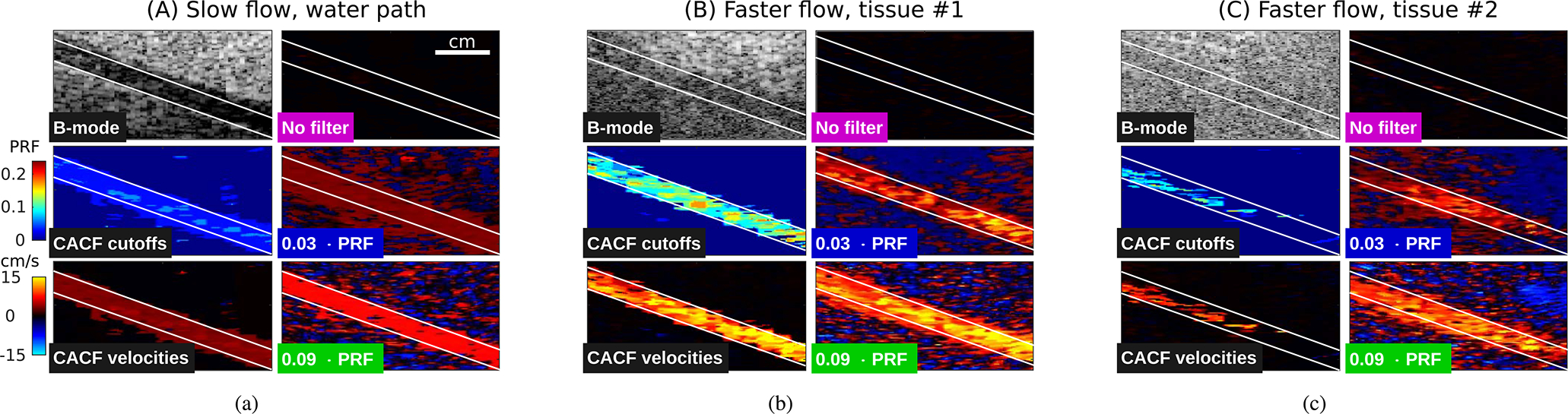
Example images sampled from A, B, and C in Fig. 3 corresponding to (a) slow flow imaged through water path and faster flow imaged through (b) tissue #1 and (c) tissue #2. Matched color flow images are shown for no filtering, conventional filtering with cutoff frequencies at *f_c_* = 0.03 and 0.09 · PRF, and CACF. B-mode images and CACF filter selections are included for reference with ROIs used for measurements in Fig. 3 outlined in white.

Fig. 4 shows example color flow images from points A, B, and C in Fig. 3. These capture conditions where notable differences can be observed between conventional and coherence-adaptive filtering. Fig. 4a shows matched images of slow flow acquired through water. Consistent with the average velocity measurements in Fig. 3a, the low cutoff filter image reveals low velocities expected for the slow flow rate setting, while the high cutoff filter image shows significant overestimation. CACF, which preferentially selects filters with lower cutoff frequencies under the conditions examined in A, produces an image with low velocities inside the vessel similar to the low cutoff filter.

Fig. 4b and Fig. 4c show the corresponding color flow images of the fastest flow rate imaged through clutter-generating tissue. Under moderate clutter levels from tissue #1, the high cutoff filter image shows high velocities inside the vessel consistent with the faster flow rate setting. Meanwhile, the low cutoff filter image shows artifactual regions of low velocity both inside and outside of the vessel. CACF, which preferentially selects filters with higher cutoff frequencies under the conditions examined in B, produces an image with high velocities inside the vessel similar to the high cutoff filter.

As clutter levels increase with tissue #2, significant degradation is observed across all filters in Fig. 4c. With conventional filtering, moving clutter results in flash artifacts outside and decreased velocities inside the vessel. This is most apparent in the low cutoff filter image, which shows severe underestimation and poor visualization of flow at both ends of the vessel. In the CACF image, large regions of the vessel show velocities close or equal to 0 cm/s. As indicated by the filter selections, this loss of velocity information is associated with a preferential selection of no filtering (*f*_*c*_ = 0 · PRF) for pixels inside the vessel.

#### 4) COLOR FLOW ARTIFACTS

**FIGURE 5.**
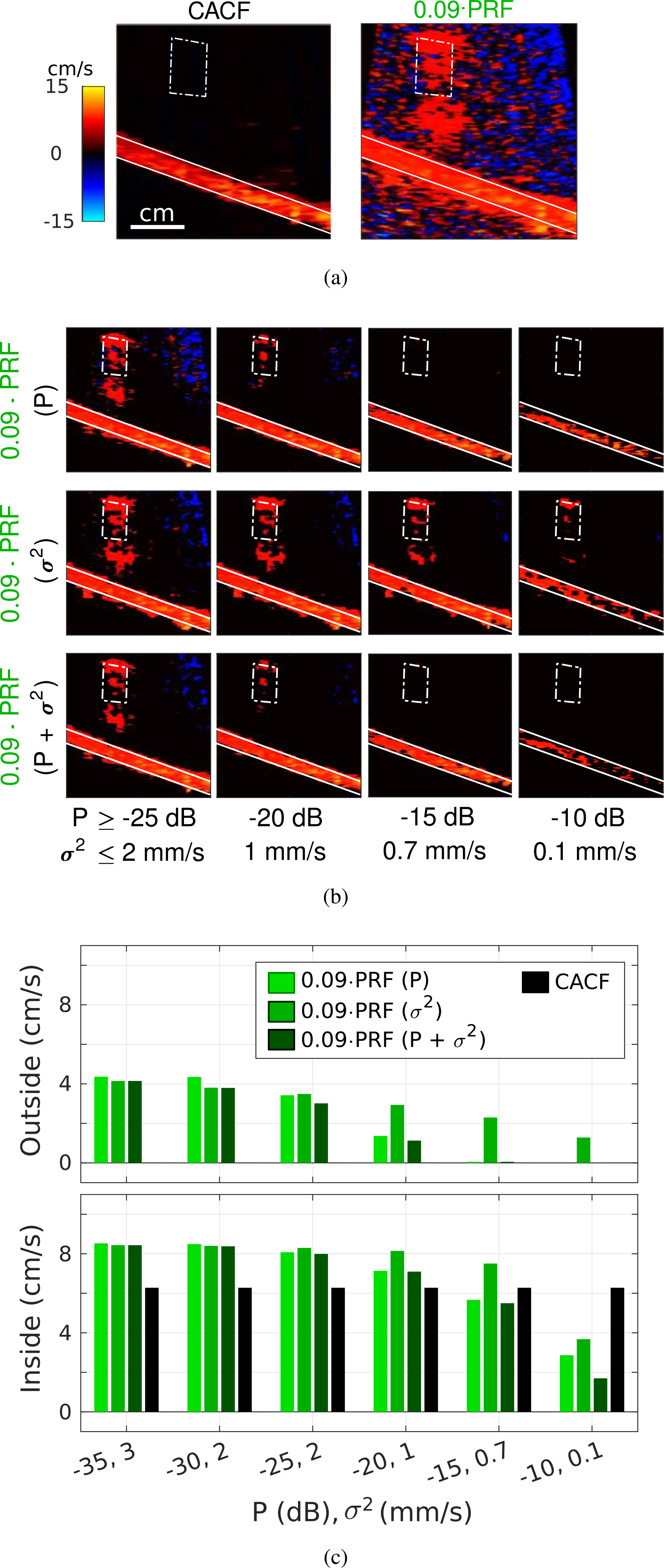
Color flow images of the CIRS 069 vessel formed with (a) conventional filtering with *f*_*c*_ = 0.09 · PRF and CACF, both without priority encoding, and (b) conventional filtering with various power (top), variance (middle), and combined power and variance (bottom) thresholds. (c) Average velocities inside and outside the vessel measured from ROIs outlined in solid and dashed white, respectively.

Figs. 5a and 5b show a series of matched color flow images formed from the same channel data acquired through tissue #1. Fig. 5a compares conventional filtering with *f*_*c*_ = 0.09·PRF and CACF without priority encoding, while Fig. 5b compares conventional filtering with priority encoding for a range of different power (P) and variance (*σ*^2^) thresholds.

Color flow images formed with conventional color flow processing reveal artifactual velocities outside of the vessel in the form of random thermal noise (jitter) and bulk motion from moving clutter (flash), neither of which are apparent in the corresponding CACF image. These artifacts can be reduced by decreasing the variance threshold or increasing the power threshold. As observed in the rightmost columns of Fig. 5b, this is associated with a concomitant loss of pixels inside the vessel as flow signal is removed along with the artifacts.

These trends are captured Fig. 5c, which plots the average velocities measured in ROIs defined inside and outside of the vessel for different P and *σ*^2^. Given that the phantom is stationary with motion confined to scatterers inside the vessel, mean velocities outside the vessel (top) are expected to approach 0 cm/s, with nonzero values indicating the presence of artifacts.

Consistent with the images in Figs. 5a and 5b, decreases in the average velocities both inside and outside of the vessel are observed for conventional processing (green) with increasing P and decreasing *σ*^2^, suggesting an intrinsic trade-off between the suppression of artifacts and the preservation of flow with priority encoding. This results in an optimal power threshold at *P* = −15 dB, which achieves complete suppression of flash artifacts (0 cm/s outside) while maintaining an average velocity inside the vessel around 6 cm/s. Variance thresholding, designed specifically to remove high variance jitter, is largely ineffective in suppressing flash artifacts, resulting in velocities outside the vessel > 0 cm/s for thresholds as low as *σ*^2^ ≤ 0.1 mm/s.

Corresponding average velocities for CACF without priority encoding are plotted in black in Fig. 5c. Results show an average velocity outside the vessel approaching 0 cm/s with an average velocity inside the vessel of 6.3 cm/s. These measurements are largely consistent with CACF images in Figs. 4a, 4b and 5a, which show clear delineation between moving scatterers inside and stationary scatterers outside the vessel.

### B. In Vivo STUDIES

#### 1) COLOR FLOW IMAGES

**FIGURE 6.**
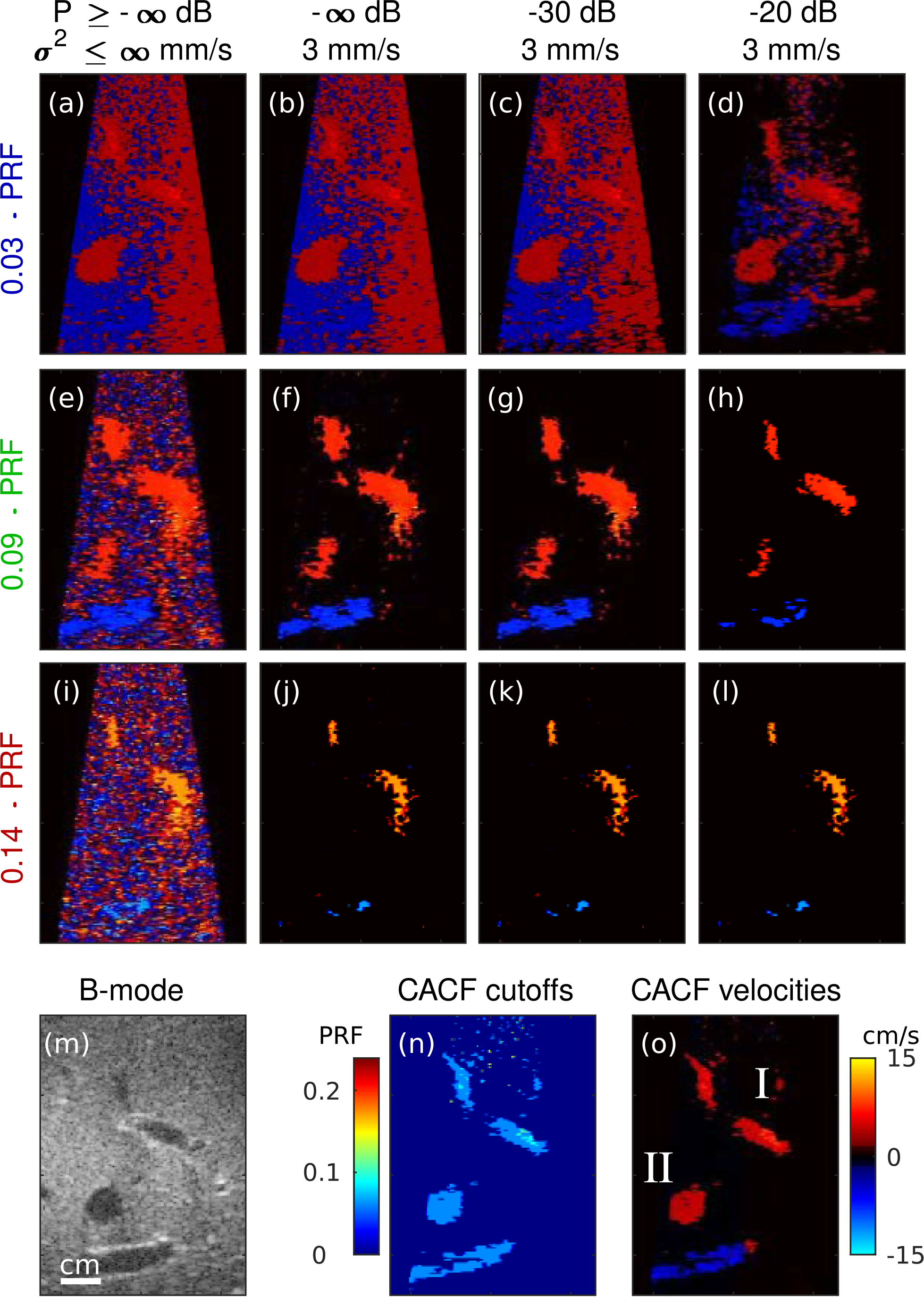
Color flow images of liver vessels formed using conventional filtering with increasing filter cutoff (top to bottom) and increasing priority thresholds (left to right). Corresponding B-mode images and images of the selected filter cutoffs and velocities with CACF are included on the bottom.

**FIGURE 7.**
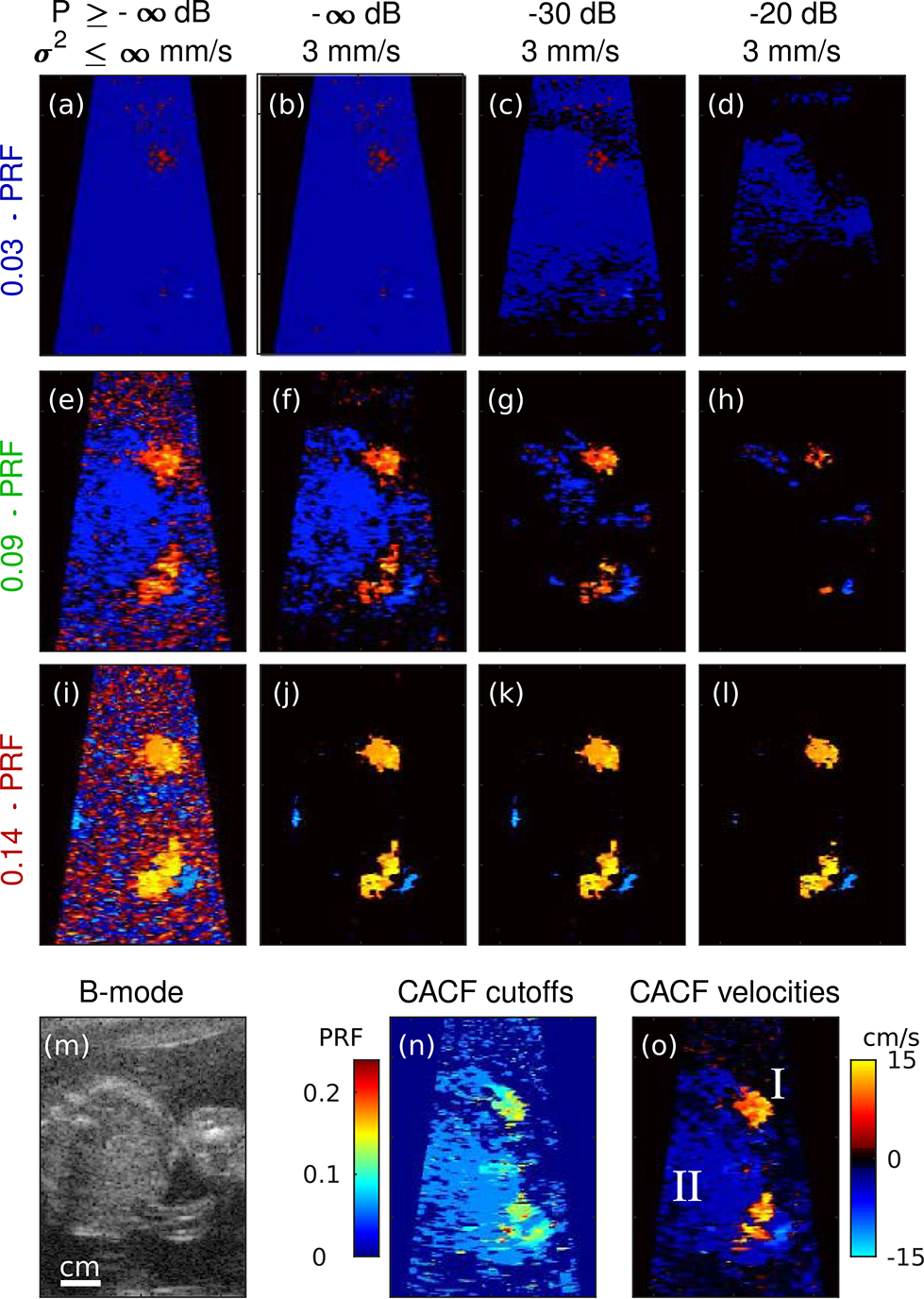
Color flow images of fetal umbilical cord and vessels formed using conventional filtering with increasing filter cutoff (top to bottom) and increasing priority thresholds (left to right). Corresponding B-mode images and images of the selected filter cutoffs and velocities with CACF are included on the bottom.

Figs. 6 and 7 show example color flow images obtained from *in vivo* liver and fetal vessels. Matched images are shown for conventional processing with increasing filter cutoff frequency (*f*_*c*_ = 0.03, 0.09, and 0.14 · PRF) from top to bottom and increasing priority encoding from left to right. Corresponding CACF velocities and filter selections without priority encoding are included at the bottom of each figure.

Fig. 6 shows color flow images of the liver with regions containing small vessels (I) and slow flow (II). Images formed with the lowest cutoff filter reveal significant flash artifacts. These artifacts can be reduced with increasing cutoff frequency and priority thresholds, but not without loss of flow signal. With higher cutoff frequencies, blood flow in both I and II is attenuated, leaving high variance jitter from thermal noise (Figs. 6e and 6i). With increased priority encoding, small vessels in I are removed before flash artifacts can be fully eliminated (Fig. 6d). Careful selection of the filter cutoff and priority thresholds in Fig. 6f can yield an optimized image capable of capturing flow in both I and II. Alternatively, a similar image can be directly obtained using CACF (Fig. 6o).

Fig. 7 shows color flow images of umbilical flow (I) in a moving fetus (II). For lower cutoff frequencies (Figs. 7a to 7d), fetal motion results in flash artifacts that overwrite the higher velocity umbilical flow. As shown in Figs. 7e and 7i, these velocities can be recovered using higher cutoff filters with stop bands wide enough to attenuate clutter from the nearby moving tissue. This is similarly achieved with CACF, which selects for higher cutoff frequencies in regions containing flow (Figs. 7n and 7o).

Note that Fig. 7o shows stable velocity estimates in not only the umbilical vessel but also the fetus itself, suggesting a potential for CACF to simultaneously capture motion from both blood and tissue. This is possible to a certain degree with conventional processing in Fig. 7f, but is made difficult by its strong dependence on filter cutoff and priority encoding which can lead to the loss of velocity information in either blood (Figs. 7a to 7d) or tissue (Figs. 7i to 7l) when tuned inappropriately.

#### 2) COLOR FLOW STABILITY

**FIGURE 8.**
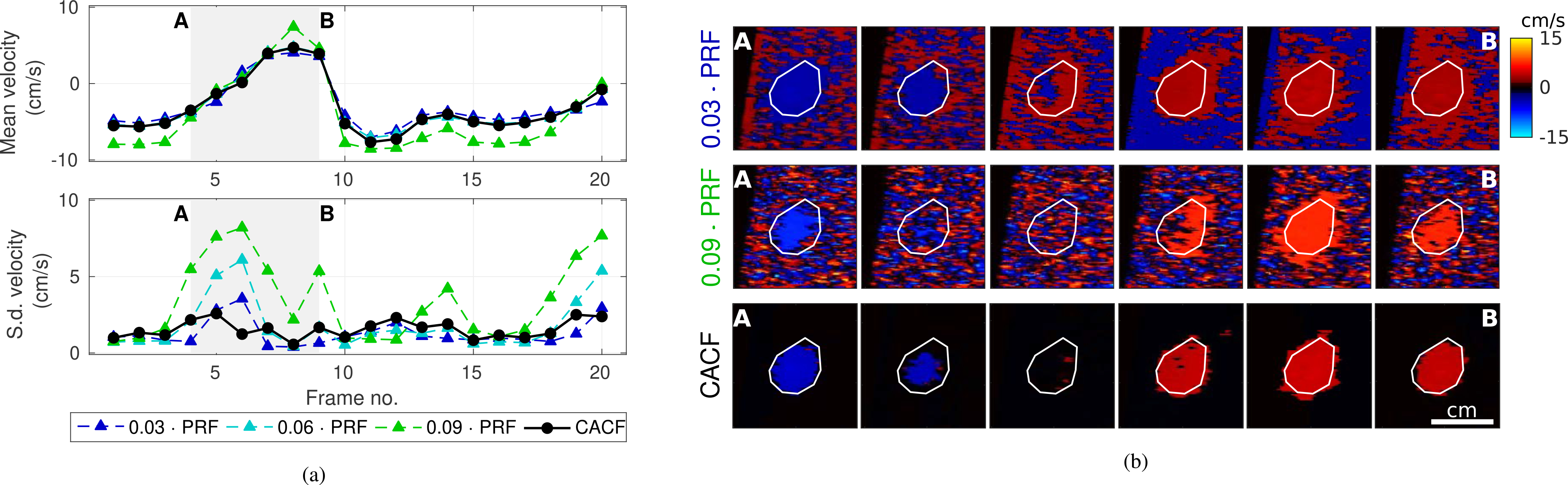
(a) Average and standard deviation of velocity estimates from multiple frames of the liver vessel in Fig. 6. Data are shown for conventional filtering with *f*_*c*_ = 0.03 and 0.09 · PRF and CACF. (b) Example images corresponding to frames A through B with ROIs outlined in white.

**FIGURE 9.**
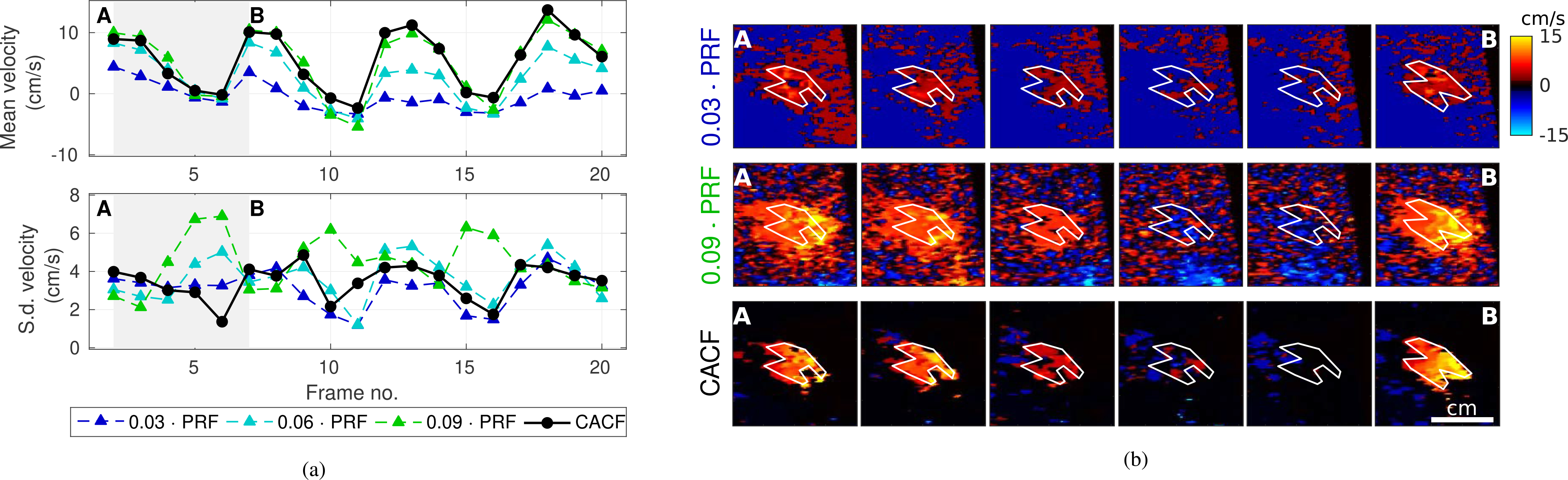
(a) Average and standard deviation of velocity estimates from multiple frames of the umbilical vessel in Fig. 6. with fetal motion. Data are shown for conventional filtering with *f*_*c*_ = 0.03 and 0.09 · PRF and CACF. (b) Example images corresponding to frames A through B with ROIs outlined in white.

Figs. 8 and 9 examine the stability of CACF in the liver vessel (II) in Fig. 6 and umbilical vessel (I) in Fig. 7, respectively. Figs. 8a and 9a compare the averages and standard deviations of velocity estimates over multiple frames of matched color flow images formed with conventional and coherence-adaptive filtering. Images from a subset of these frames are included for reference in Figs. 8b and 9b.

For the liver vessel in Fig. 8, CACF shows time-varying velocities that closely match the velocities measured with conventional filtering. With regards to spatial stability, measurements of standard deviation for CACF are comparable to those of the best-case conventional clutter filter (*f*_*c*_ = 0.03 · PRF). For conventional filters, particularly those with higher cutoff frequencies, increased standard deviations are observed in frames with lower velocities where flow signal is removed leaving residual jitter. This is not observed with CACF, which maintains relatively low standard deviations across all 20 frames (Fig. 8b).

For the umbilical vessel in Fig. 9, average velocities with CACF are observed to track closely with velocities from higher cutoff filters, which more aggressively attenuate clutter generated by fetal motion. Unlike higher cutoff filters, however, the recovery of flow from moving clutter with CACF is not observed with a concomitant increase in jitter. As in Fig. 8a, standard deviations with CACF in Fig. 9a are on par with the lower standard deviations observed with conventional filtering with *f*_*c*_ = 0.03 and 0.06 · PRF.

## IV. DISCUSSION

### A. COLOR FLOW ACCURACY

In phantom studies, velocity measurements obtained using a low cutoff filter under clutter-free conditions were used as an approximation for the ground truth flow rate. While an absolute comparison of measured velocities to the actual ground truth velocities would be far more compelling, the derivation of such values is challenging, and when performed incorrectly, can be confounded by numerous factors including the Doppler angle, flow pump calibrations, beam geometry, and spatial and temporal nonuniformities. These measured velocities therefore serve as an imperfect but necessary reference to assess the performance of different filters operating under different imaging conditions. Of the conditions examined in this study, they are expected to most appropriately capture the ideal, bias-free behavior given the wide filter passband and lack of spectral content from moving clutter or non-flow signal.

Given this standard of comparison, CACF is observed to maintain close to ideal velocity estimation across a range of flow rates under clutter-free and moderately cluttered conditions. As shown by Figs. 4a and 4b, this is largely attributed to the appropriate selection of low cutoff filters under clutter-free conditions to minimize the attenuation of slow flow and high cutoff filters under cluttered conditions to suppress slow-moving reverberation and/or off-axis clutter. The result is an increased dynamic range of velocity estimation, capable of providing more accurate visualization of both low and high velocity flow without the manipulation of filter cutoffs and/or priority thresholds.

Improvements in dynamic range are similarly observed under *in vivo* imaging conditions. In liver vessels in Fig. 6, CACF provides direct visualization of slow flow and small vessel branches that are otherwise overwritten by flash artifacts in filters with too low of a cutoff frequency or removed in filters with too high of a cutoff frequency. For fetal images in Fig. 7, this translates to the ability to image both high velocity blood flow and low velocity tissue motion. Filters selected by coherence feedback to optimize the visualization of liver vessels (Fig. 6) and umbilical flow (Fig. 7) span a wide range, with higher cutoff frequencies selected in Fig. 7 in comparison to Fig. 6. Such results suggest that a conventional filter optimized for imaging in one clinical setting may be inadequate for another. While ground truth velocities are unavailable *in vivo*, phantom results suggest that CACF images likely provide a more accurate representation of *in vivo* blood flow and tissue motion compared to conventional images.

It should be noted that the simultaneous measurement of blood and tissue velocities as shown in Fig. 7o represents a unique property of CACF. This has potential utility in applications such as echocardiography where the measurement of both blood and tissue dynamics are clinically-relevant [29]. On the other hand, in applications focusing solely on the characterization of blood, the appearance of tissue velocities may be less desirable and likened to flash artifacts. In such cases, CACF, as implemented in this study, may be ill-suited, and future work should explore the addition of amplitude-and/or velocity-dependent processing in combination with CACF to provide this added level of blood and tissue signal discrimination.

For the 3 kHz PRF used in this study, physiological motion in the liver from respiration may not always induce appreciable changes in average phase across the ensemble. For this reason, we believe that the ~0 cm/s velocities in the liver tissue in Figures 6 and 8 are expected. Meanwhile, fetal motion is highly dynamic and can produce large variations in tissue velocity as shown in Figure 7.

### B. COLOR FLOW STABILITY

While the pixel-wise selection of clutter filters is expected to introduce an additional source of variance in velocity estimation, results in Figs. 8 and 9 suggest comparable temporal and spatial stability between CACF and conventional filtering. Under conditions both with and without tissue motion, CACF velocities are well-behaved over time with standard deviations that approach those of the best-case conventional filter. The increased dynamic range of CACF furthermore allows for stable velocity estimation and decreased standard deviations in frames with slow flow that are otherwise dominated by jitter with conventional filtering. Such results support the ability for CACF to not only improve velocity estimation accuracy, but do so without sacrificing temporal or spatial stability.

### C. COLOR FLOW ARTIFACTS

As shown in both phantom and *in vivo* images, the simultaneous measurement of coherent signal from both tissue and blood scatterers with CACF leads to a reduction in artifacts commonly encountered in conventional color flow imaging. While tissue echoes are removed by conventional filters, leading to the measurement of velocities from residual thermal noise, reverberations, and off-axis clutter, CACF preserves tissue echoes to derive more stable velocity estimates in regions without blood. This reduces the dependence on priority encoding and other forms of post-processing, all of which rely on the appropriate selection of manually-tuned thresholds and parameters. As shown by Fig. 5, color flow images formed with CACF alone were able to outperform matched images formed with both conventional filtering and priority encoding with regards to not only velocity estimation accuracy, but also the severity of color artifacts.

It should be noted that many methods for priority encoding and color processing exist on commercial systems which are far more sophisticated than the power and variance thresholds applied in this study. Results in this study therefore serve as a demonstrative, rather than exhaustive comparison of CACF to conventional color flow processing. Nonetheless, all of such post-processing methods are expected to have similar limitations related to manual parameter tuning that may be alleviated with CACF pre-processing.

### D. LIMITATIONS AND FUTURE WORK

While many scenarios examined in this study show improvements in color flow image quality with CACF, there remain instances where the proposed method fails.

#### 1) LOW SPATIAL COHERENCE

In regions containing high levels of weakly-coherent clutter, thermal noise, and/or focusing errors, measurements of SLSC are expected to be low across the entire filter bank. As characterized in previous studies, low values of SLSC are associated with increased variance, which in CACFleads to an increase in the variance of filter selections and output velocity estimates [24]. This is readily observed in the amniotic fluid in Fig. 7 and the near-field in Fig. 6, where low-amplitude signals and focusing errors are expected to result in SLSC values approaching zero.

**FIGURE 10.**
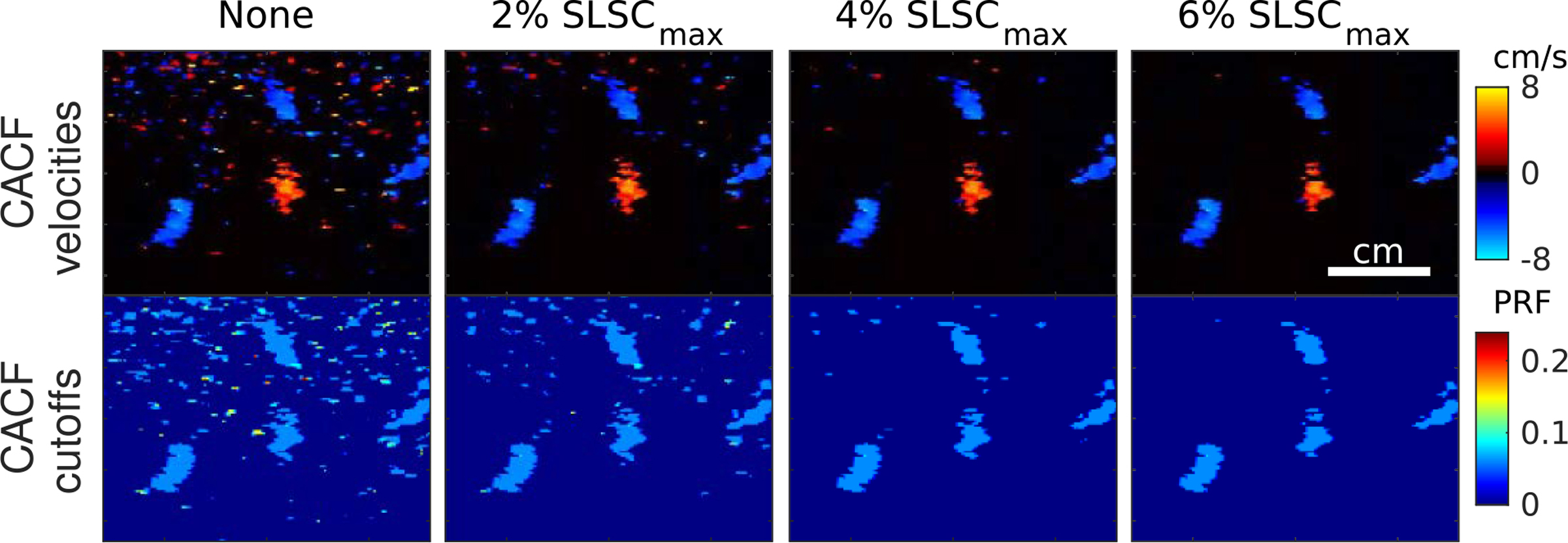
Color flow images of small liver vessels formed using CACF with increasing levels of coherence thresholding applied going from left to right.

Besides conventional post-processing methods, one potential mitigation for this degradation would be to apply some form of thresholding in order to reject pixels with low spatial coherence. Fig. 10 provides an example of such an approach applied to liver vessels located in the near-field. In this example, a pixel was assigned no clutter filter if its maximum SLSC value across all filters fell below 2, 4, and 6% of the theoretical maximum SLSC in speckle. With small amounts of thresholding, CACF images are able to show reduced variance with little to no change in regions containing flow.

#### 2) CORRELATED CLUTTER

Degradation in CACF is also observed with correlated clutter, which can arise from a variety of sources including nearby off-axis scattering and coherent reverberations. As shown in previous simulations studies, under conditions with high clutter levels and/or low signal-to-noise ratio, these forms of correlated clutter can appear more coherent than blood, leading to the selection of filters containing slow-moving clutter and a decrease in the accuracy of CACF velocities [24].

In the cluttered phantom images from tissue sample #2 (Fig. 4c),), incoherent acoustic clutter and thermal noise are expected to more significantly degrade the coherence of blood compared to partially coherent, but higher amplitude clutter. This directly explains the selection of no filtering by CACF in Fig. 4c, which in this case, maximizes coherence by preserving the correlated off-axis clutter overwriting lower amplitude blood inside the vessel. While this represents a notable limitation of the proposed method, it should be noted that such behaviour is present only under conditions which represent challenging imaging scenarios for conventional color flow processing as well.

A possible mitigation for the loss of flow with CACF would be to apply a non-uniform weighting to the SLSC values measured across different filters, thereby tuning coherence feedback to preferentially select a specific subset of clutter filters. For instance, SLSC values at the output of the first filter could be weighted by *w*_1_, the second by *w*_2_, and so on. Such a scheme would allow filters containing small amounts of flow to be selected over those containing large amounts of correlated clutter. More advanced filtering schemes and their associated trade-offs should be investigated in future work to improve the performance of CACF under these known failure modes.

### E. COMPUTATION TIME

For *M* channels, *K* slow-time samples, and *F* clutter filters, the expected number of floating point operations (FLOPs) required for CACF is approximately $O(FKM^{2}+FK^{2}M\;+\;FKM\text{)}$, where *FKM*^2^, *FK*^2^*M*, and *FKM* represent the number of operations required for coherence calculation, clutter filtering, and channel summation, respectively. This is in comparison to the $O\left(KM+K^{2}\right)$ FLOPs required for conventional clutter filtering, which consists of a channel sum followed by a single filtering operation. In the example of a 64-channel system with 14 slow-time samples and 8 clutter filters, this amounts to a roughly 520× slowdown with CACF over conventional filtering assuming serial CPU implementations similar to those used in this study.

While this may seem prohibitive, it should be noted that CACF is highly parallelizable as SLSC estimates can be independently computed not only for each pixel, but also each slow-time sample and filter. Additionally, algorithmic changes can be leveraged to further minimize computational load. This includes sparsing the pixels used for adaptive filter selection as well as the number of channels and/or slow-time samples used to compute the SLSC in each filter. Given previous demonstrations of real-time imaging using coherence-based techniques [30]–[32], the above shows promise for efficient implementations of CACF with parallel, high throughput computing devices such as GPUs.

## V. CONCLUSION

In this study, CACF was evaluated under realistic imaging conditions in phantom and *in vivo*. In phantom experiments with clutter-generating tissue, CACF was shown to reduce velocity estimation bias from slow-moving clutter or the attenuation of flow signal, resulting in an increase in the dynamic range of velocity estimates compared to conventional filtering. The simultaneous measurement of both blood and tissue motion by CACF was shown to reduce the severity of flash artifacts and jitter, decreasing the need for carefully tuned priority thresholds. In liver and fetal imaging, these properties translated to the improved visualization of blood vessels, which were otherwise overwritten by moving clutter or removed with suboptimal filtering and/or priority encoding. Improved color flow image quality was observed without significant decreases in temporal or spatial stability due to the pixel-wise selection of clutter filters. While failure modes in the current implementation of CACF were identified, a number of proposed solutions show promise to mitigate these limitations with minimal overhead. Together, these findings support the feasibility of CACF as a method to optimize color flow image quality without the operator dependence of conventional color flow processing.

## Figures and Tables

**TABLE 1.** Clutter measurements.

	Thickness	B-mode contrast	LOC

Water	N/A	−14.0 dB	0.75
Tissue #1	~2 cm	−11.1 dB	0.45
Tissue #2	~4 cm	−4.4 dB	0.36
